# Population burst propagation across interacting areas of the brain

**DOI:** 10.1152/jn.00066.2022

**Published:** 2022-11-02

**Authors:** Yu Chen, Hannah Douglas, Bryan J. Medina, Motolani Olarinre, Joshua H. Siegle, Robert E. Kass

**Affiliations:** ^1^Machine Learning Department, Carnegie Mellon University, Pittsburgh, Pennsylvania; ^2^Neuroscience Institute, Carnegie Mellon University, Pittsburgh, Pennsylvania; ^3^Department of Statistics & Data Science, Carnegie Mellon University, Pittsburgh, Pennsylvania; ^4^MindScope Program, Allen Institute, Seattle, Washington; ^5^Department of Computer Science, University of Central Florida, Orlando, Florida

**Keywords:** cross-region communication, multiple spike trains, neuropixels, point process, trial-to-trial variation

## Abstract

For many perceptual and behavioral tasks, a prominent feature of neural spike trains involves high firing rates across relatively short intervals of time. We call these events “population bursts.” Because during a population burst information is, presumably, transmitted from one part of the brain to another, burst timing should reveal activity related to the flow of information across neural circuits. We developed a statistical method (based on a point process model) of determining, accurately, the time of the maximum (peak) population firing rate on a trial-by-trial basis and used it to characterize burst propagation across areas. We then examined the tendency of peak firing rates in distinct brain areas to shift earlier or later in time, together, across repeated trials, and found this trial-to-trial coupling of peak times to be a sensitive indicator of interaction across populations. In the data we examined, from the Allen Brain Observatory, we found many very strong correlations (95% confidence intervals above 0.75) in cases where standard methods were unable to demonstrate cross-area correlation. The statistical model introduced cross-area covariation only through population-level trial-dependent time shifts and gain constants (values of which were learned from the data), yet it provided very good fits to data histograms, including histograms of spike count correlations within and across visual areas. Our results demonstrate the utility of carefully assessing timing and propagation, across brain regions, of transient bursts in neural population activity, based on multiple spike train recordings.

**NEW & NOTEWORTHY** We developed a novel statistical method for identifying coordinated propagation of activity across populations of spiking neurons, with high temporal accuracy. Using simultaneous recordings from three visual areas we document precise timing relationships on a trial-by-trial basis, and we show how previously existing techniques can fail to discover coordinated activity in cases where the new approach finds very strong cross-area correlation.

## Graphical abstract

**Figure:**
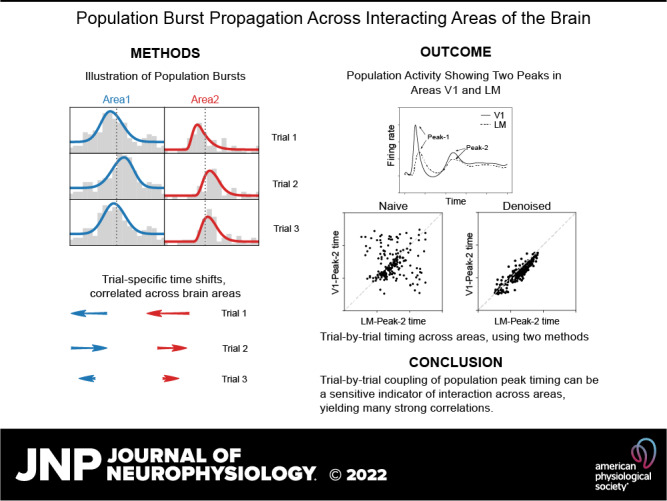

## INTRODUCTION

Recent advances in electrophysiological recording technologies have dramatically increased the number of neurons and brain regions that can be recorded in a single experiment (1–3), offering new opportunities for identifying functional interactions among multiple populations of neurons. Peristimulus time histograms (PSTHs) are a simple and useful way to compare the relative timing of neural activity across regions and are therefore widely used, but because they aggregate data across trials, PSTHs (or smoothed PSTHs) cannot capture trial-to-trial variation and co-variation (4–6). Studies of trial-to-trial covariability in populations of spiking neurons most often consider spike counts in relatively wide windows (7–13) and even the papers that model individual spike times have typically ignored the precise timing of firing rate fluctuations (14, 15). Implicit in these and many other physiological investigations, however, is the assumption that behaviorally relevant information is transmitted across parts of the brain through transient bursts of activity in neural populations. These population bursts are sometimes evoked by a stimulus, but they could also be associated with a movement or other behavior, and they are sometimes called phasic as opposed to tonic (16, 17). If population bursts are indeed important, their timing should reveal coordinated activity; on a trial-by-trial basis, the time of a burst in one population should be related to the time of a corresponding burst in a downstream population. We chose to examine the time of maximal (peak) firing rate because it is advantageous as an object of statistical estimation: by definition, relatively large numbers of spikes occur near that time, so that trial-to-trial covariation in the timing of population bursts (in our study, stimulus-evoked responses), could be a sensitive indicator of cross-population interaction. We developed a model, and associated methodology, focused on a trial-specific time of peak firing rate in each of several neural populations, and the trial-to-trial covariation of these times across populations. We report the resulting multiple-population spiking model, and fitting algorithm, together with an analysis of data recorded simultaneously from three areas of the mouse visual system.

**Figure 1.**
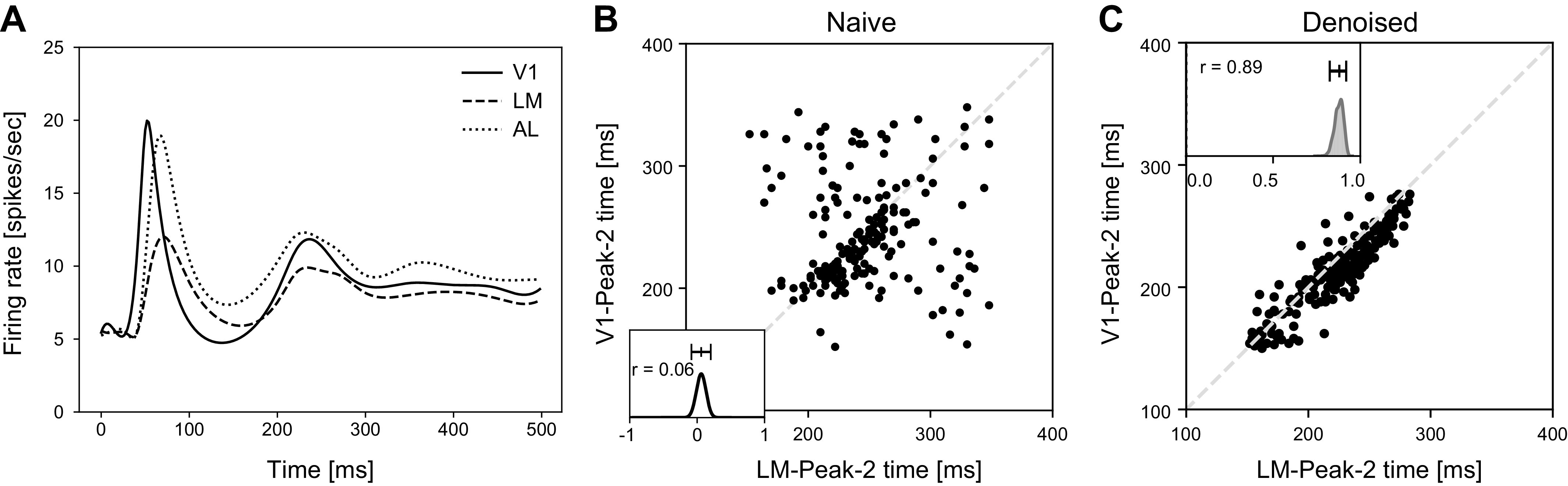
Illustration of trial-to-trial correlation in peak timing across populations. *A*: smoothed population PSTHs in response to full-field drifting gratings for mouse visual areas V1, LM, and AL. The three population peristimulus time histograms (PSTHs) have similar features: the first peak appears at around 60 ms after the onset of the stimulus (time zero), and the second peak appears at around 250 ms after onset. *B* and *C*: comparison between a naive method and results from our Interacting Population Rate Function (IPRF) model on recovery of Peak-2 timing within primary visual cortex (V1) and lateral medial visual area (LM). Peak-2 time refers to the time of maximal firing rate calculated differently for the naive method and the IPRF model. The results in *B* and *C* are based on the same subset of active neurons. In both *B* and *C* each dot, representing one of 195 trials (15 trials in each of 13 experimental conditions), displays the estimated times of the second peak in regions V1 and LM. The naive method in *B* finds the peak of a smoothed population PSTH for each area. No relationship between the peak times in V1 and LM is visible; the correlation is small and the 95% confidence interval (−0.09, 0.20) makes it statistically indistinguishable from zero. In *C*, strong covariation appears. The estimated correlation is 0.89 and the 95% confidence interval is (0.82, 0.92) (based on the posterior mode and 0.025 and 0.975 quantiles). AL, anterolateral visual area. Details about this figure are in *Materials and Preprocessing*.

The data we have analyzed [available from the Allen Brain Observatory (18)] are from the primary visual cortex (V1), the lateral medial visual area (LM), and the anterolateral visual area (AL), three regions that lie at distinct hierarchical processing stages while being tightly interconnected (2, 19). To help motivate our approach, Fig. 1 provides a preview of a striking result and its context. We considered the two large peaks in population firing rate, representing evoked responses, seen in Fig. 1*A*; we analyzed the times of maximal firing rate relative to the onset of drifting gratings stimuli, along with the overall (time-averaged) firing rate. A dual-peaked sensory response has been observed in a variety of model systems. In the visual cortex, the second peak is correlated with the perception of a stimulus in humans (20, 21) and macaques (22). In the mouse somatosensory cortex, the second peak has been shown to depend on feedback from motor areas (23), and disrupting this peak impairs the detection of tactile stimuli (23, 24). Thus, while the first peak likely reflects the feedforward signal propagation up the sensory hierarchy, the second peak is strongly affected by top-down feedback signals. As activity propagates across multiple brain areas on a trial-by-trial basis, consistency in the relative timing of these peaks would reveal close coordination of areas as an essential feature of the sensory response. For these three visual areas, one might expect to see such close coordination for the first peak, but effects of feedback are less well documented. It is therefore less clear what to expect for the second peak. Our results show that the coordination across areas is very strong for the second peak and, in terms of our assessments, it is stronger for the second peak than for the first.

In Fig. 1*A*, the trial-averaged time of the second peak in V1 seems to be only slightly ahead of the time of the second peak in LM, to which V1 is connected anatomically. A simple way to examine the trial-by-trial covariation in the timing of the V1 and LM second peaks would be to smooth the two population PSTHs on each trial, i.e., compute a trial-by-trial version of the curves in Fig. 1*A*, and find the time of maximal firing rate from each of those curves. This is not helpful, however, because, as illustrated in Fig. 1*B*, the resulting peak times are very noisy. Figure 1*B* shows no consistent relationship, on a trial-by-trial basis, between the timing of the second peak in V1 and the timing of the second peak in LM (the correlation is small and not statistically different than zero), which, if true, would be surprising. The method we have developed greatly reduces the effects of noise in the population spike trains, leaving behind the very strong trial-to-trial correlation seen in Fig. 1*C* [posterior mode 0.89, and 95% confidence interval (CI) (0.82, 0.92)].

The method’s denoising of time lag estimation and correlation is achieved by *1*) introducing a statistical model (a point process model) for the population spike trains that include trial-dependent time shifts for each of the two peaks, *2*) allowing the shapes of the population firing rate curves to vary across experimental conditions, and *3*) focusing on condition-specific subsets of neurons that participate in the population. These elements are all incorporated into what we call an Interacting Population Rate Function (IPRF) model. The method has roots in Ref. 6, which showed how point process models could fit trial-to-trial covariation in neural spike trains, and Refs. 25 and 26, which documented the ability of statistical models to denoise spike count correlation; it also builds on the large body of work on point process modeling of spike trains (27, 28, 29, 30, 31 chapter 19). Point process models are, essentially, spike count models using time bins chosen to be so small that at most only one spike is likely to occur in any one bin, and the count (0 or 1) in each bin is assumed to be Poisson with a bin-specific firing rate. (Mathematically, they occur in continuous time and we have just described the discrete-time approximation; see Ref. 31 chapter 19.) Although the resulting spike count distributions in broader intervals need not themselves be Poisson (due to possible statistical dependence among the firing rates across time bins), the stochastic variation of point process models accounts for the Poisson-like noise in spiking behavior that is universally observed in vivo, and they are typically implemented using standard generalized regression methods (31 chapter 19). Point processes have long been considered the natural mathematical framework for modeling spike trains. In the work reported here, we did not model individual spike trains. Rather, the IPRF model describes the behavior of each population spike train formed by merging the spike times from neurons within that population, and the resulting covariation across populations. The model introduces correlation only through trial-dependent population shifts in time and trial-dependent population gain constants (see *Model Overview and Specification*). Nonetheless, it recapitulates many aspects of spike train behavior, including the distribution of spike count correlations both within and across brain areas (Fig. 9*B* and Supplemental Fig. S15; all Supplemental Material is available at https://www.doi.org/10.5281/zenodo.7024023).

One benefit of denoising is to reveal relationships, like that displayed in Fig. 1*C*, that would otherwise be masked. Another is to improve the precision of estimates. For example, when we used the naive method to estimate the lag of LM Peak-2 time behind V1 Peak-2 time, we obtained a wide 95% CI of (−5.3, 10.9) ms, which is not statistically differentiated from zero; using, instead, the method we developed we found this lag to have 95% CI of (6.2, 13.0) ms. In *Data Analysis* (see Fig. 5), we give additional estimates of the time lags among activity peaks in the three areas and we show disaggregated population firing rate curves that differ from the population PSTHs in Fig. 1*A*: the PSTH representations of firing rate in Fig. 1*A* are distorted by averaging across conditions and blurring across time, due to the merging of trials without accounting for their different shifts in time (see also Supplemental Fig. S10). On the other hand, when we average the fitted firing-rate curves across trials, within conditions, the fitted curves match the within-condition PSTHs well (Fig. 9 and Supplemental Fig. S14).

In addition to denoising of peak timing, the method introduced here has two other purposes. The first is to describe the functional diversity and specialization of neurons that are relevant to cross-area coordinated activity (32–34). We estimate the proportion of recorded neurons that participate in this kind of population activity, and we indicate their cortical depths. The second further purpose is to describe multivariate relationships, specifically, pairwise assessment of covariation for two brain areas conditionally on (statistically “holding fixed”) activity in the third area. We can thus supplement correlation with partial correlation. We discuss such results, and their interpretation, in *Data Analysis.*

## METHODS

### Model Overview and Specification

**Figure 2.**
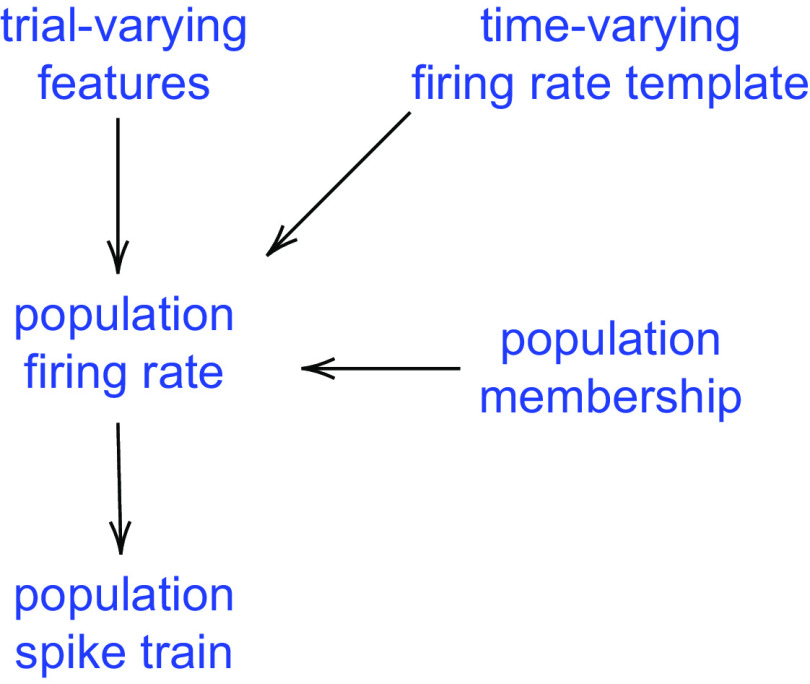
Outline of the Interacting Population Rate Function (IPRF) model for a single area and a specified condition. On a given trial, each population spike train is driven by its population firing rate, which combines a time-varying firing-rate template with trial-varying features. Only a subset of neurons recorded within the brain area are used, and this subpopulation is determined by a population membership probability.

**Figure 3.**
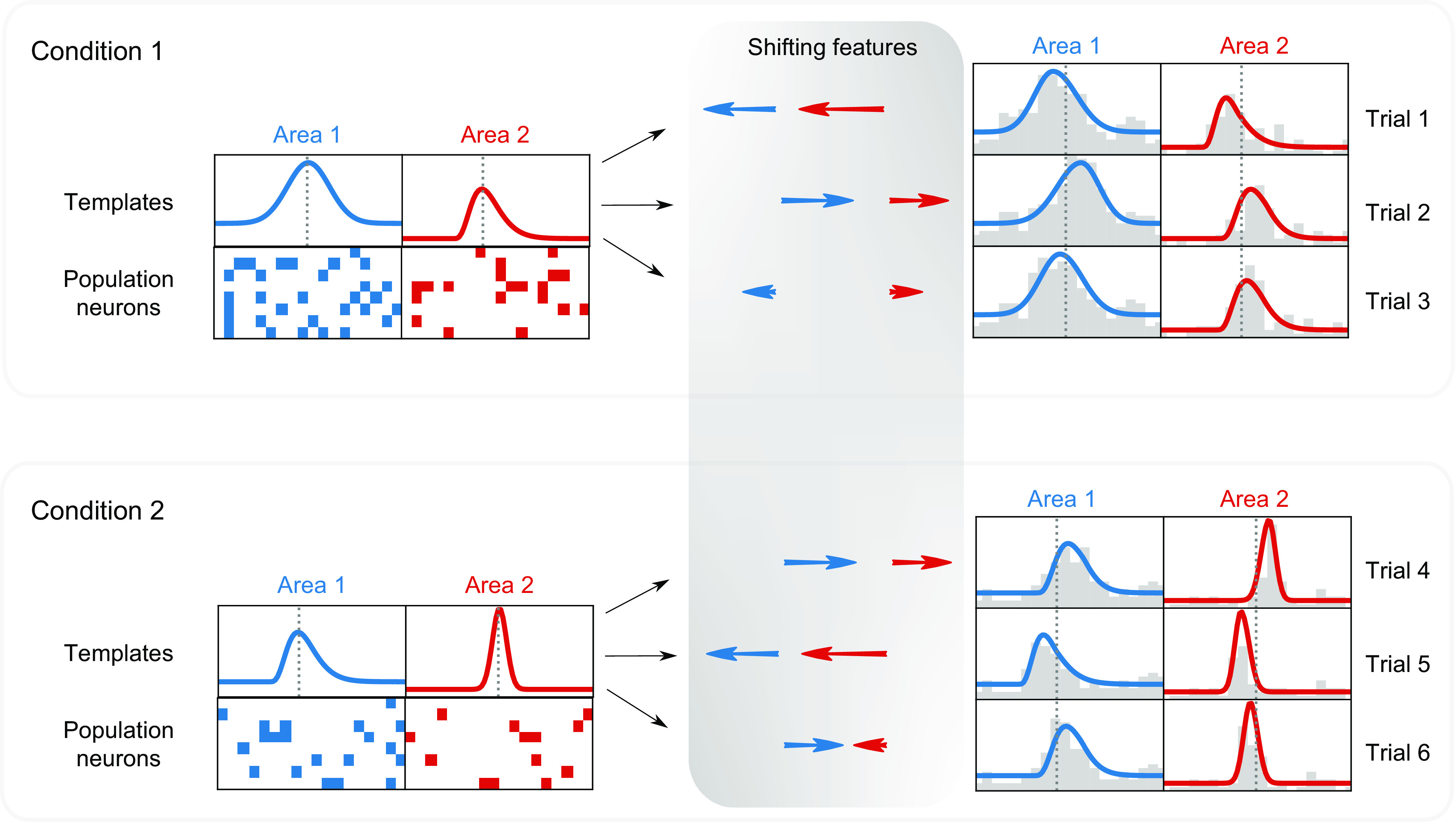
Toy illustration of Interacting Population Rate Function (IPRF) model. For each of two conditions, shown in separate large boxes, population firing-rate functions are displayed on the right side of the diagram [together with corresponding population peristimulus time histograms (PSTHs)] for two areas across three trials. The blue (*area 1*) and red (*area 2*) firing-rate functions use data-driven templates (displayed on the left side of the diagram), based on some subpopulation of recorded neurons (displayed by the grid-like plots on the left side showing the positions of active neurons along the multi-electrode array). The diagram displays different populations within each area under conditions 1 and 2. The template shifts (displayed in the middle of the diagram) vary with each trial to produce the trial-dependent firing-rate functions. The shifts for *areas 1* and *2* are different, but may be highly correlated. Not shown explicitly here is the additional possibility that each trial has its own gain constant. In this illustration there is only one peak in each template, rather than two peaks.

**Figure 4.**
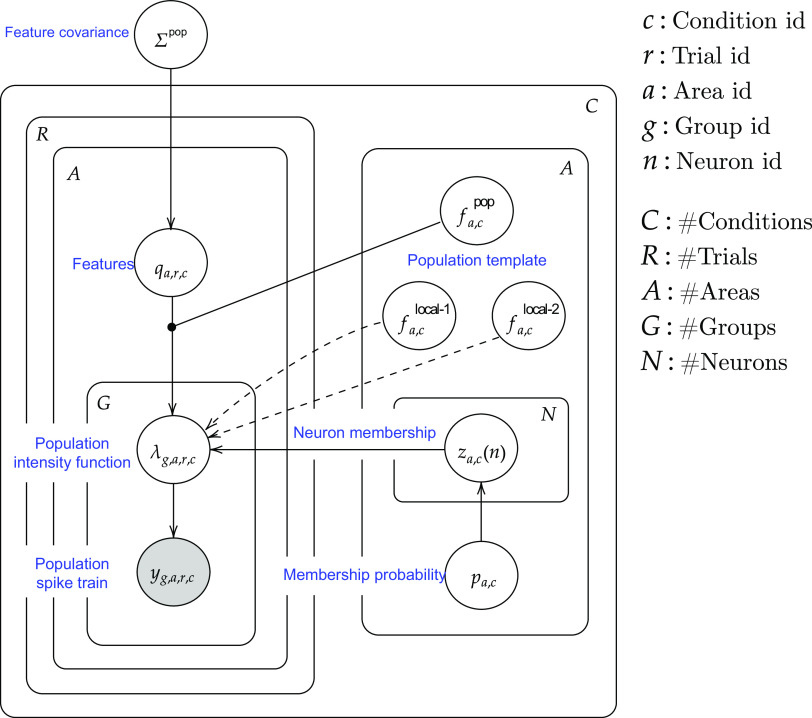
Graphical display of the Interacting Population Rate Function (IPRF) statistical model. As explained in the text, on a given trial, for a given area and condition, the data are spike trains. For a given area and condition each neuron is assigned to one of three populations (for a given area and condition) and the population spike trains *y* are modeled as point processes with intensities λ. Only one of the three populations is of interest, namely, the interacting subpopulation having template *f*^pop^. The covarying features *q* are combined with the population template *f*^pop^, which appears (probabilistically) in the intensity. The indicators *z* and probabilities *p* define the assignment of neurons to the templates $f^{\text{pop}},\;f^{\text{local-1}}$, and *f* ^local-2^ (see *Eq. 2*). Although the assignment is “soft” in the sense that it is probabilistic, our results in Fig. 8 show that for the mouse visual data the probabilities of assignment to the interacting subpopulation are almost always above 0.9 or below 0.1. The templates are learned from the data using nonparametric smoothing (*Eq. 10*).

The IPRF model describes, for each of several brain areas, a time-varying population firing rate function (template) having key parameters (features) that vary across trials to control the timing of the response, based on an appropriate subpopulation of neurons. A high-level sketch of this model structure for a single area, under a particular condition, is shown in Fig. 2, with an additional visual description provided by Fig. 3. Central to the scientific use of the IPRF model is the cross-area trial-to-trial variation and covariation of the features (the shifts in peak time) that appear at the top of Fig. 2 and in the shaded region forming the middle column of Fig. 3. In Fig. 4 these features are given the variable name *q* (or *q_a_*_,_*_r_*_,_*_c_* for area *a* on trial *r* in condition *c*). Our main data analytic results concern the timing of the peaks in the template (representing a model-based average across trials) and the way the values *q_a_*_,_*_r_*_,_*_c_* make the population peak firing rates vary across trials, and vary together across areas.

The complete structure of the IPRF model appears in Fig. 4, in the graphical model form used frequently in statistics and machine learning (the full mathematical specification is given in the next paragraph). Technically, it is a latent variable model with both discrete and continuous latent variables; it could also be called a hierarchical model in the sense of Bayesian statistics (though, for simplicity, the priors are not shown in the diagram). Notice, first, that neurons, indexed by *n*, come from areas, indexed by *a*, and repeated trials, indexed by *r*, are within conditions, indexed by *c*. Conceptually (beginning at the bottom of the diagram), the population spike train formed from the collective spike times from all the neurons within that population is a point process determined by a population firing rate (intensity) function. The intensity function for a population in area *a* under condition *c* depends on the collection of neurons, in that condition, that participates in cross-area population activity: neuron *n* has probability *p*_0_(*n*) of belonging to the interacting population having firing rate intensity function written in terms of $f_{a,c}^{\text{pop}}$, which we call a template. Population membership is signified by the variable *z_a_*_,_*_c_*(*n*) (taking values labeled *g* for “group”), where *n* = 1,…,*N*. Collectively, the *z_a_*_,_*_c_*(*n*) values form the membership vector *z_a_*_,_*_c_* (see *Eq. 2*). As the subscripts indicate, the membership vectors are specific to an area (in which particular neurons are located) and can change with the condition. The template is also specific to area *a* and condition *c*. The membership vectors and templates are learned from the data (the templates are penalized splines having 100 knots; see *Eq. 10*). The template $f_{a,c}^{\text{pop}}$ is subjected to trial-specific morphing via time-warping, which allows shifts in the times of the two peaks we focus on (as shown in Fig. 3 for a single peak). These two peak times are two of the features which, together with a gain constant, form the feature vector labeled *q*. The diagram shows the feature vector being combined with the firing rate template when it becomes an input to the population intensity function. The features capture trial-to-trial variation, while the template is the intensity corresponding to the mean (across trials) of the features. With probability 1 – *p*_0_(*n*), neuron *n* is not in the interacting population and, instead, joins one of two alternative populations driven by different firing rate function templates, either $f_{a,c}^{\text{local-1}}$, which is time-varying or $f_{a,c}^{\text{local-2}}$, which is constant in time, according to probabilities *p*_1_(*n*) and *p*_2_(*n*) (where *p*_0_(*n*) + *p*_1_(*n*) + *p*_2_(*n*) = 1 for all *n*). In Fig. 4, these three scalars for all *N* neurons are collected into a vector labeled membership probabilities. The template functions $f_{a,c}^{\text{pop}}$ and $f_{a,c}^{\text{local-1}}$ are fitted with penalized splines. Finally, the covariation of the nine-dimensional feature vector (3 parameters for each of 3 areas) is summarized in a covariance matrix.

The more precise specification of the model begins with the population spike trains and corresponding intensity function,

(*1*) $$y_{g,a,r,c}(t)\;|\;{\text{λ}}_{g,a,r,c}(t),z_{a,c}∼\text{ Poisson}(N_{g,a,c}\cdot {\text{λ}}_{g,a,r,c}(t))$$

(*2*) $$\begin{array}{l} \log\;{\text{λ}}_{g,a,r,c}(t)\;|\;q_{a,r,c,},f_{a,c}^{\text{pop}},f_{a,c}^{\text{local-1}},f_{a,c}^{\text{local-2}}\\ =\;\{\begin{array}{ll} f_{a,c}^{\text{pop}}({\text{φ}}_{a,r,c}^{-1}(t\;|\;q_{a,r,c}^{\text{peak-1}},q_{a,r,c}^{\text{peak-2}}))+q_{a,r,c}^{\text{gain}}, & \text{if }g=\text{pop}\\ f_{a,c}^{\text{local-1}}(t), & \text{if }g=\text{local-1}\\ f_{a,c}^{\text{local-2}}(t), & \text{if }g=\text{local-2}, \end{array} \end{array}$$

where *g* is the group index, which can be “pop,” “local-1,” “local-2,” *y_g_*_,_*_a_*_,_*_r_*_,_*_c_*(*t*) is the population spike train of group *g* in area *a* trial *r* and condition *c*, *N_g_*_,_*_a_*_,_*_c_* is the number of neurons for which *z_a_*_,_*_c_*(*n*) = *g*, φ is the population template time-warping function, specified in terms of the feature vector *q_a_*_,_*_r_*_,_*_c_*, and the templates are defined in terms of spline bases *B* and coefficient vectors β,

(*3*) $$f_{a,c}^{\text{pop}}\;|\;{\text{β}}_{a,c}^{\text{pop}}=\;B{\text{β}}_{a,c}^{\text{pop}},$$

(*4*) $$f_{a,c}^{\text{local-1}}\;|\;{\text{β}}_{a,c}^{\text{local-1}}=\;B{\text{β}}_{a,c}^{\text{local-1}}.$$

(*5*) $$f_{a,c}^{\text{local-2}}\;|\;{\text{β}}_{a,c}^{\text{local-2}}=\;1{\text{β}}_{a,c}^{\text{local-2}}.$$

The template group membership indicator *z* is categorical,

(*6*) $$z_{a,c}(n)\;|\;p_{a,c}∼\text{ categorical}(p_{a,c}),$$

where the three components of each vector *p_a_*_,_*_c_* sum to 1. [Categorical(*p*) has the same probabilities as multinomial(*n*, *p*) with *n* = 1.]

To complete the model, we define the feature vector, its probability distribution, and the time-warping function. We consider three features in each region, denoted $q_{a,r,c}^{\text{gain}},\;q_{a,r,c}^{\text{peak-1}}$, and $q_{a,r,c}^{\text{peak-2}}$, representing the two peak times (more specifically, the trial-specific deviations of the peak times from those of the trial-invariant template $f_{a,c}^{\text{pop}}$) and a gain constant; the gain constant allows the integrated firing rate (integrated across the whole time interval, which controls the expected number of spikes) to vary across areas, conditions, and trials. For other data applications, different areas could have different numbers of features corresponding to, for example, two peaks in one area and a single peak in a different area. For a given feature vector, the time-warping function ${\text{φ}}_{a,r,c}:[0,T]\mapsto [0,T]$ modifies a template *f* from a function of time *f*(*t*) to the same function of warped time *f*(φ^−1^(*t*)). Also, in $f_{a,c}^{\text{pop}}$ the constant $q_{a,r,c}^{\text{gain}}$ is added to the time-warped template. We assume the warping function is piecewise linear with the following join-points (also called knots, or landmarks): (0, 0), (*t*_1,_*_L_*, *t*_1,_*_L_*), $\left(t_{\text{peak-1}},t_{\text{peak-1}}+q_{a,r,c}^{\text{peak-1}}\right)$, (*t*_1,_*_R_*, *t*_1,_*_R_*), (*t*_2,_*_L_*, *t*_2,_*_L_*), (*t*_peak-1_, *t*_peak-1_+$q_{a,r,c}^{\text{peak-1}}$), (*t*_2,_*_R_*, *t*_2,_*_R_*), (*T*, *T*). Here, the domain of peak-1 is [*t*_1,_*_L_*, *t*_1,_*_R_*], meaning that *t*_1,_*_L_* and *t*_1,_*_R_* are chosen so that the interval spans (roughly) the times at which the firing rate profile defines the peak. The time-warping function maps the time of peak-1 from *t*_peak-1_ to $t_{\text{peak-1}}+q_{a,r,c}^{\text{peak-1}}$. Peak-2 is treated similarly. Piecewise linearity forces linear interpolation of time from the beginning (or end) of the peak range to the peak. The times *t*_1,_*_L_*, *t*_peak-1_, *t*_1,_*_R_*, *t*_2,_*_L_*, *t*_peak-2_, *t*_2,_*_R_* are fitted at the initialization step. Finally, all features from all areas are stacked together to form a vector *q*_·,_*_r_*_,_*_c_* (a 9-dimensional vector using 3 features from 3 areas in our case) which is assumed to follow a normal distribution across trials (and with a fixed distribution across conditions).

(*7*) $$q_{\cdot ,r,c}\;|\;\Sigma^{\text{pop}}∼\;N(0,\Sigma^{\text{pop}}).$$

Note that *q_a_*_,_*_r_*_,_*_c_* is modeling the trial *r* deviations from the population template $f_{a,c}^{\text{pop}}$, which represents (in the sense of the model) the interacting population firing rate shape averaged across trials. This is the reason for setting the mean of *q_a_*_,_*_r_*_,_*_c_* to be the zero vector.

Aside from the template functions $f_{a,c}^{\text{pop}}$ and $f_{a,c}^{\text{local-1}}$ (each having one smoothing parameter determined by cross-validation), the free parameters are in the matrix Σ^pop^ (which has 45 parameters as a 9 × 9 symmetric matrix) and the membership probability vectors *p_a_*_,_*_c_* (which has *N* × *C* = 117). In addition, the expectation–maximization (EM) algorithm and Gibbs sampling algorithms estimate the continuous latent variables *q_a_*_,_*_r_*_,_*_c_* and discrete latent variables *z_a_*_,_*_c_*(*n*).

### Model Fitting and Uncertainty Assessment

We have used maximum likelihood for initial fitting, and then Bayesian inference via posterior distributions to assess uncertainty. The hierarchical structure of the model lends itself to application of the EM algorithm and Gibbs sampling. These share a common algorithmic structure, shown in Algorithm 1, with EM and Gibbs sampling each providing their own definitions of the “update.” Here, we have used the so-called “hard EM” rather than standard EM, substituting conditional posterior modes for expectations, in the E-step, as in K-means clustering (35, p. 354). In *Gibbs Sampling Details* we give details of our Gibbs sampling update implementation. We omit EM updating because it is standard, and easily implemented once the structure of the model and resulting likelihood function are understood. In practice, we first complete an initialization step, then we run EM to get maximum likelihood estimates and use those results as initialization for running Gibbs sampling to get posterior distributions based on specified priors.

#### Template functions.

The template functions $f_{a,c}^{\text{pop}}$ and $f_{a,c}^{\text{local-1}}$ are splines with equally spaced knots (in our data analysis we used 100 knots across the 500-ms interval). They are fitted, in the update steps, using penalized likelihood, with second-derivative penalties as in smoothing splines (31 section 15.2.5; 36). The smoothing parameter is found using fivefold cross-validation. In the case of Poisson processes fitting, intensity estimation is equivalent to probability density estimation (see Ref. 31 section 19.2.2) and, in density estimation, smoothing splines adapt to varying smoothness (37). Thus, in this context, we expect penalized splines to fit well (see also related results in Ref. 36 and Supplemental Curve Fitting).

The purpose of $f_{a,c}^{\text{local-1}}$ is to improve on the constant firing rate provided by $f_{a,c}^{\text{local-2}}$ in fitting neurons that are irrelevant to cross-population activity.

Nothing in the formal specification of the model prohibits equality of $f_{a,c}^{\text{pop}}$ and $f_{a,c}^{\text{local-1}}$, which could create poor convergence of fitting procedures. In practice, however, fits to $f_{a,c}^{\text{pop}}$ and $f_{a,c}^{\text{local-1}}$ are clearly distinguished during initialization.

#### Initialization.

We initialized the model by first sorting neurons and assigning them to the three groups (“pop,” “local-1,” and “local2”) according to their activity strengths being “high,” “medium,” or “low” firing rate, based on spike count during the 500 ms following stimulus onset, after merging across conditions and trials, letting 25%, 50%, and 25% of the neurons fall into these groups. These three groups and proportions were used to initialize the neuron membership identifiers *z_a_*_,_*_c_*(*n*) and associated probabilities *p_a_*_,_*_c_*. The templates $f_{a,c}^{\text{pop}}$ and $f_{a,c}^{\text{local-1}}$ (i.e., the spline basis coefficients) were initialized from the “high” and “medium” groups using penalized splines, with penalty constant chosen through simulation experiments; $f_{a,c}^{\text{local-2}}$ were fitted as constants from the “low” group. After obtaining the initial templates, we performed visual screening to verify that each $f_{a,c}^{\text{pop}}$ had a strong and clear two-peak pattern. These simple heuristics always produced visually satisfactory results. From $f_{a,c}^{\text{pop}}$ we found the time-warping landmarks *t*_1,_*_L_*, *t*_peak-1_, *t*_1,_*_R_*, *t*_2,_*_R_*, *t*_peak-2_, *t*_2,_*_R_*. The feature vectors *q_a_*_,_*_r_*_,_*_c_*, whose elements represent deviations from the template $f_{a,c}^{\text{pop}}$, were initialized to (0, 0, 0).

#### Prior distributions.

In our implementation we have used the prior distributions

(*8*) $$\Sigma^{\text{pop}}\;|\;\Psi_{0},\nu_{0}∼\text{ IW}(\Psi_{0},\nu_{0})$$

(*9*) $$p_{a,c}\;|\text{ α}∼\text{ Dirichlet}(\text{α})$$

with hyperparameters Ψ_0_, ν_0_, α. To make the prior on Σ diffuse, we set *ν*_0 = 3_ *A* + 1. We used Ψ_0 = 2_Φ_0_ where Φ_0_ is a diagonal matrix with the square roots of the diagonal elements being equal to the approximate range of *q* based on data from a different animal. We set α = 5·**1**. Additional information may be found in Supplemental Priors.

#### Correlation, partial correlation, and regression.

The estimate of Σ^pop^, together with samples from its posterior distribution, immediately provide the correlations, partial correlations, and regression estimates among the quantities of interest *q* (also see Supplemental Properties of Multivariate Normal Distribution). More specifically, first, if *V* (*X*) = Σ for an *m*-dimensional random vector *X*, all $\left(\begin{array}{c} m\\ 2 \end{array}\right)$ correlations are obtainable from Σ; second, this means that an estimate of Σ produces estimates of the correlations, and samples from the distribution of Σ produce samples from the distributions of those correlations; third, if we partition as *X* = (*X*^(1)^, *X*^(2)^, *X*^(3)^) with *X*^(1)^ being univariate, standard formulas (again using the elements of Σ) also provide estimates of, together with samples from the distribution of *1*) the regression of *X*^(1)^ on *X*^(2)^ and *2*) when *X*^(2)^ is also univariate, the partial correlation of *X*^(1)^ and *X*^(2)^ conditionally on *X*^(3)^. Thus, the method provides estimates and uncertainties for any regression or partial correlation we wish to examine.

### Gibbs Sampling Details

Here we provide details of the Gibbs sampling updates in Algorithm 1. Our code is available online at www.github.com/AlbertYuChen/IPRF.

a)  Updating $f_{a,c}^{\text{pop}},\;{\text{β}}_{a,c}^{\text{pop}}$. The full conditional log posterior is
(*10*) $$\begin{array}{l} \begin{array}{l} \log\;p(f_{a,c}^{\text{pop}}|\ldots )=\log\;p(B{\text{β}}_{a,c}^{\text{pop}}|\ldots )=\sum_{r}\ell (y_{g,a,r,c}({\text{φ}}_{a,r,c}(t)),N_{g,a,c}\cdot \exp\;\{f_{a,c}^{\text{pop}}+q_{a,r,c}^{\text{gain}}\})+\text{η}P(f_{a,c}^{\text{pop}})+\text{const} \end{array}\\ P(f)=-N_{g,a,c}\cdot R_{c}\cdot {\text{β}}^{\text{pop},T}\Omega {\text{β}}^{\text{pop}}\\ \Omega_{ij}=\int f_{i}^{\left(2\right)}(x)f_{j}^{\left(2\right)}(x)dx, \end{array}$$
  where $f_{i}^{\left(2\right)}$ is the second derivative of the *i*th cubic spline basis element, $N_{g,a,c}\cdot R_{c}$ is the total number of spike trains, and *ℓ*(*y*,λ) denotes the log-likelihood for a discretized point process with spike train *y* and intensity λ. The function $f_{a,c}^{\text{pop}}$ is fitted using third-order smoothing spline basis *B* in *Eq. 3*). We used 100 knots equally spaced in the 500-ms window (so there are 102 basis elements). The sequence of spike counts in 2 ms time bins is *y*. The penalty ηP on the coefficients is designed for smoothness in the same spirit as the smoothing spline (38, section 5.4). In Gibbs sampling, the penalty becomes the log prior density, where the prior is normal. The smoothing parameter η is tuned using cross-validation. Details are discussed in Supplemental Curve Fitting. The gain $q_{a,r,c}^{\text{gain}}$ is used as the offset. Metropolis–Hastings sampling is embedded in this step of Gibbs sampling. Letting β′ be a candidate sample and β*^m^* a sample from the last iteration *m*, the proposal distribution is
$$\tilde{q}(\text{β}\prime |{\text{β}}^{m})=N({\text{β}}^{m},0.05Q),$$
  where *Q* is the inverse Hessian matrix for β of the posterior at the mode. We estimate *Q* in the initialization step and hold it fixed subsequently. We follow the suggestion in Ref. 39, Chapter 12, of setting the scale coefficient of the proposal distribution covariance matrix to be 5.76/*d* ≈ 0.05, where *d* is the dimension of the covariance matrix. This balances the trade-off between exploration and rejection. The acceptance ratio on average is kept between 0.2 and 0.8. The acceptance ratio of Metropolis–Hastings sampling method is
$$a=\min\;\left(1,\frac{p(\text{β}\prime )\tilde{q}({\text{β}}^{m}|\text{β}\prime )}{p({\text{β}}^{m})\tilde{q}(\text{β}\prime |{\text{β}}^{m})}\right)$$
b)  Updating $f_{a,c}^{\text{local-1}},\;f_{a,c}^{\text{local-2}},\;{\text{β}}_{a,c}^{\text{local-1}}$ and ${\text{β}}_{a,c}^{\text{local-2}}$. The full conditional log posterior is similar to that of $f_{a,c}^{\text{pop}}$. We have
(*11*) $$\begin{array}{l} \log\;p(f_{a,c}^{\text{local-1}}|\ldots )=\log\;p(B{\text{β}}_{a,c}^{\text{local-1}}|\ldots )\\ =\sum_{r,g=\text{local-1}}\ell (y_{g,a,r,c},\;N_{g,a,c}\cdot \exp\;\{f_{a,c}^{\text{local-1}}\})+\text{η}P(f_{a,c}^{\text{local-1}})+\text{const}. \end{array}$$
  The calculation for $f^{\text{local-2}}$ is similar, but simplified because it is a constant times the **1** vector.
c)  Updating $q_{a,r,c}=(q_{a,r,c}^{\text{gain}},q_{a,r,c}^{\text{peak-1}},q_{a,r,c}^{\text{peak-2}})$. We have
log p(qa,r,c|…)=ℓ(yg,a,r,c(φa,r,c(t)), Npop,a,c· exp {fa,cpop+qa,r,cgain})+log N(qa,r,c;0,Σpop)+const.
  The time-warping function φ*_a_*_,_*_r_*_,_*_c_* is parameterized by $q_{a,r,c}^{\text{peak-1}},\;q_{a,r,c}^{\text{peak-2}}$, see *Model Overview and Specification*. $f_{a,c}^{\text{pop}}$ is given and is treated as the offset. The landmark positions *t*_peak-1_ and *t*_peak-2_ are determined in the initialization using grid search and are not updated in subsequent fitting.Metropolis–Hastings sampling is nested in this step of Gibbs sampling. Letting *q*′ be a candidate sample and *q^m^* the sample from the last iteration at step *m*, for trial *r*, condition *c* and all areas at once (denoted by subscript *A*) a sample is drawn from the proposal distribution
$$\tilde{q}({q\prime }_{A,r,c}|q_{A,r,c}^{m})=N(q_{A,r,c}^{m}-0.5\cdot \bar{q_{A,c}^{m}},0.05Q)\cdot I_{\text{clip}\_\text{region}}(q\prime_{A,r,c}),$$
  where $\overset{\text{¯}}{q_{A,c}^{m}}$ is the mean of $q_{A,r,c}^{m}$ over all trials and $I_{\text{clip}\_\text{region}}$ truncates so that the first two components of the proposal stay within the domains of peak-1 and peak-2 (see time-warping before *Eq. 7*).Subtracting a multiple of $\bar{q_{A,c}^{m}}$ from $q_{A,r,c}^{m}$ moves the proposal mean closer to satisfying $\bar{q_{A,c}^{m}}=0$, which may be recognized as an identifiability constraint. In principle, the prior on *q_a_*_,_*_r_*_,_*_c_* forces identifiability but, because we used a diffuse prior on Σ, samples can drift with increasingly large variances. The proposal we used has lower acceptance rates at this step, but solves the drift problem and produces well-behaved sample trace plots.
d)  Updating *z_a_*_,_*_c_*(*n*).
$$z_{a,c}(n)∼\text{Categorical}(p(z_{a,c}(n)=g|\ldots )),\;g\in \{\text{pop},\text{local-1},\text{local-2}\}.$$
  Here, $p(z_{a,c}(n)=g|\ldots )$ is the probability that *z_a_*_,_*_c_*(*n*) belongs to subpopulation category g∈{pop,local-1,local-2}, which is specified as follows:
$$\begin{array}{l} \log\;p(z_{a,c}(n)|\ldots )=\sum_{r}\log\;p(y_{n,a,r,c}|z_{a,c}(n),q_{a,r,c},f_{a,c}^{\text{pop}},f_{a,c}^{\text{local-1}},f_{a,c}^{\text{local-2}})\;\\ +\log\;p(z_{a,c}(n)|p_{a,c})+\text{const}\\ =\log\;p(z_{a,c}(n)|p_{a,c})+\text{const}+\\ \sum_{r}\{\begin{array}{ll} \ell (y_{n,a,r,c}({\text{φ}}_{a,r,c}(t)),\;\exp\;\{f_{a,c}^{\text{pop}}+q_{a,r,c}^{\text{gain}}\}), & \text{if }z_{a,c}(n)=\text{pop}\\ \ell (y_{n,a,r,c},\exp\;\{f_{a,c}^{\text{local-1}}\}), & \text{if }z_{a,c}(n)=\text{local-1}\\ \ell (y_{n,a,r,c},\exp\;\{f_{a,c}^{\text{local-2}}\}), & \text{if }z_{a,c}(n)=\text{local-2}. \end{array} \end{array},$$
  where *y_n_*_,_*_a_*_,_*_r_*_,_*_c_* is the spike train of individual neurons indexed by *n*.
e)  Updating *p_a_*_,_*_c_*.
$$p_{a,c}|\ldots ∼\text{Dirichlet}(N_{\text{pop},a,c}+\text{α},\;N_{\text{local-1},a,c}+\text{α},\;N_{\text{local-2},a,c}+\text{α}).$$
  After conditioning on $z_{a,c}(n),\;p_{a,c}$ becomes independent of the rest of the variables or data. $N_{\text{pop},a,c},\;N_{\text{local-1},a,c},\;N_{\text{local-2},a,c}$ count the total number of neuron memberships *z_a_*_,_*_c_*(*n*) in each subgroup in area *a* in condition *c*.

a)  Updating Σ^pop^. We draw samples from the Inverse-Wishart distribution
$$\begin{array}{l} p(\Sigma^{\text{pop}}|\ldots )=\text{IW}(\tilde{\Psi },\tilde{\nu })\\ \tilde{\nu }=\nu_{0}+RC\\ \tilde{\Psi }=\Psi_{0}+q^{T}q \end{array},$$
  where Ψ_0_, ν_0_ are hyperparameters. The bold **q** ∈ ℝ ^*RC*×3^ is a stacked matrix of features *q_a_*_,_*_r_*_,_*_c_*. Each column represents a feature, and each row represents features for a trial. *RC* is the total number of *q*. *R* is the number of trials for each condition, and *C* is the number of conditions. (We discuss the selection of Ψ_0_, ν_0_ in Supplemental Priors.)

### Materials and Preprocessing

We applied our method to the publicly available Allen Brain Observatory-Visual Coding Neuropixels data set (2). It uses multiple high-density extracellular electrophysiology probes to simultaneously record spiking activity from a wide variety of areas in the mouse brain, especially the cortex. The animals were head-fixed and were passively presented with visual stimuli. The details of the experimental setup can be found in Refs. 2 and 18. In each session, the mouse is exposed to a variety of stimulus types, such as natural movies, flashes, Gabor filters, drifting gratings, etc. Our paper uses drifting gratings because it has many repeated trials, the stimuli are simple, the trials are long and it can strongly elicit neural responses. The drifting gratings (type drifting_gratings in the data set) have 40 conditions which are combinations of 8 different orientations (0°, 45°, 90°, 135°,180°, 225°, 270°, 315°, clockwise from 0° = right-to-left) and 5 different temporal frequencies (1, 2, 4, 8, 15 Hz). The spatial frequency is 0.04 cycles/degree and the contrast is 80%. The stimulus for each condition is repeated 15 times. A trial lasts for 3 s with 2 s stimulus and 1 s gray screen. The sequence of the conditions is randomly ordered. The baseline condition has 30 trials with a gray screen. The number of neurons in visual cortical areas recorded by one probe ranges, roughly, from 40 to 100. Usually, six probes are recorded at the same time. The data set assigns unique identities for all properties. For example, a condition is labeled by stimulus_condition_id, a trial is labeled by stimulus_presentation_id, one experiment session is labeled by ecephys_session_id. In this paper, we refer to those identities directly.

We screened the animals and conditions based on whether the regions of interest were recorded and whether the neurons have strong responses, as the time-warping features depended on the peaks of the curves. For example, session 754829445 did not record LM and AL regions. In session 746083955, the activity of AL was too weak and was almost the same as baseline activities (trials without visual stimulus), so AL was not able to provide any useful information. The preprocessing was done in three steps:

1)  Check if the target regions are recorded by any probes.
2)  Select the top 50% neurons with the largest spike counts.
3)  Select the conditions with strong fluctuating responses. We first calculate the total variation (40, section 6.3.3) of kernel-smoothed (Gaussian kernel with standard deviation 10 ms) group PSTH for each condition and each region using the neurons in step 2. Next, sort the conditions by the sum of total variation of all regions in descending order. Then select the top conditions. The cutoff is done by visual check where the last condition has clear two-peak patterns.

We analyzed mouse session 798911424 using 13 drifting gratings conditions. Each condition had 15 repeated trials. The details of the conditions are listed in Supplemental Table S1. The results for V1, LM, and AL, include 94, 89, and 78 neurons, respectively. Spike trains were binned in 2 ms.

In Fig. 1*A*, each curve represents activity aggregated across 600 trials: there were 15 trials in each combination of 8 orientations and 5 frequencies. Only the most active 50% of neurons with large spike counts were selected. The curves were fitted using regression with splines. The displayed distribution of the correlation estimator and its confidence interval in Fig. 1*B* was calculated using the asymptotic normal distribution of the Fisher *z*-transformed correlation, as in Ref. 31, section 12.4.3. The correlation and confidence interval in Fig. 1*C* was obtained from the posterior of the correlation, implemented as described in *Gibbs Sampling Details.*

### Goodness-of-Fit Assessment

We check the fits of the IPRF model by performing KS tests, comparing with PSTHs, and comparing with spike count correlation histograms. Results will be shown in *Data Analysis* and Supplemental File. The KS plots were based on the time-rescaling theorem (41, 42), but the KS statistics are subject to some bias because the inter-spike intervals are limited by the trial length, so only short intervals can be observed. To get the results shown in Supplemental Fig. S18, we corrected for the bias by adjusting the null distribution of transformed inter-spike intervals according to the trial length.

## RESULTS

This section reports selected findings from our data analysis and from the simulation study designed to check the likely accuracy of data analytic conclusions.

### Data Analysis

Implementation of the IPRF model discovers many potentially interesting relationships involving the trial-to-trial distributions of peak times, peak coupling across brain areas, population firing rate profiles, and descriptions of neuron diversity. Here, we have chosen to discuss only a few illustrative results. Some others appear in the Supplemental Material.

#### Peak timing and its variability.

**Figure 5.**
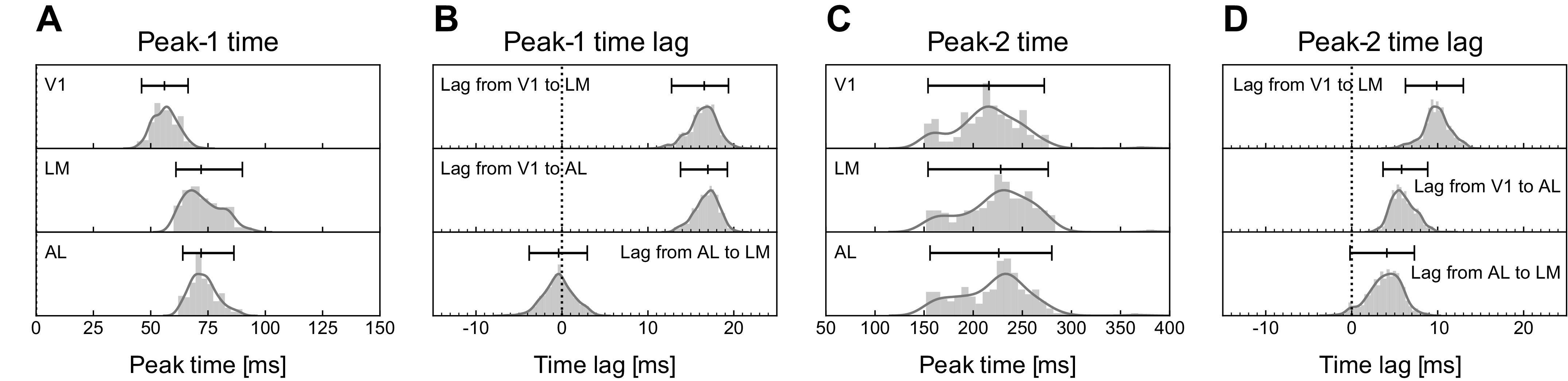
Distributions, across trials, of peak timing and of time lags between areas. Histograms of the estimated peak times and time lags are shown together with median and 0.025 and 0.975 quantiles. The histogram in *A* was formed from the collection (across trials) of Peak-1 times estimated for each trial, and the other histograms were created similarly. *A*: the histogram of estimated Peak-1 times for primary visual cortex (V1) is centered at 57 ms poststimulus onset with 95% of values falling in the range (46, 66) ms; for lateral medial visual area (LM) and anterolateral visual area (AL) the corresponding times are 68 (60, 90) ms and 70 (64, 86) ms. *B*: the histograms show that V1 leads LM by a median of 17 ms with 95% of trials having lead times in the range (13, 19) ms; V1 leads AL by 17 (14, 19) ms; and AL and LM are roughly simultaneous, with the lag from AL to LM being −0.4 (−3.8, 2.9) ms. *C*: histograms of estimated Peak-2 times show that Peak-2 times are much more variable than Peak-1 times. For V1 the Peak-2 times are centered at 216 ms with 95% of trials having times in the range (154, 272) ms; for LM and AL the values are 232 (154, 277) ms and 233 (156, 278) ms. *D*: the Peak-2 time lags between areas are much more precise than the times themselves, and lags are much smaller than those for Peak-1: V1 leads LM by 9.6 ms with 95% of trials falling in the range (6.2, 13.0) ms; V1 leads AL by 5.5 (3.6, 8.8) ms; AL leads LM by 4.6 (−0.2, 7.3) ms.

Figure 5 displays the distribution across trials of estimated Peak-1 and Peak-2 times, together with their time lags across areas. The variability across trials in Peak-1 timing (Fig. 5*A*) is substantial, but perhaps not very surprising: if we were to think of the difference between the 0.975 quantile and the 0.025 quantile as representing very roughly 4 standard deviations, the trial-to-trial standard deviation for Peak-1 times in V1 would be ∼5 ms. The Peak-2 times (Fig. 5*C*) are very much larger. The analogous calculation gives a corresponding standard deviation for Peak-2 times in V1 of ∼30 ms. Interestingly, the lags between areas for Peak-2 have roughly the same amount of variability as the lags between areas for Peak-1, and they are only about half as long. Presumably this indicates there are additional inputs (feedback being plausible) that contribute to Peak-2 timing. It is also noteworthy that for Peak-2 times, AL tends to be earlier than LM, whereas for Peak-1 times there is no such tendency.

#### Peak-2 correlation and partial correlation.

**Figure 6.**
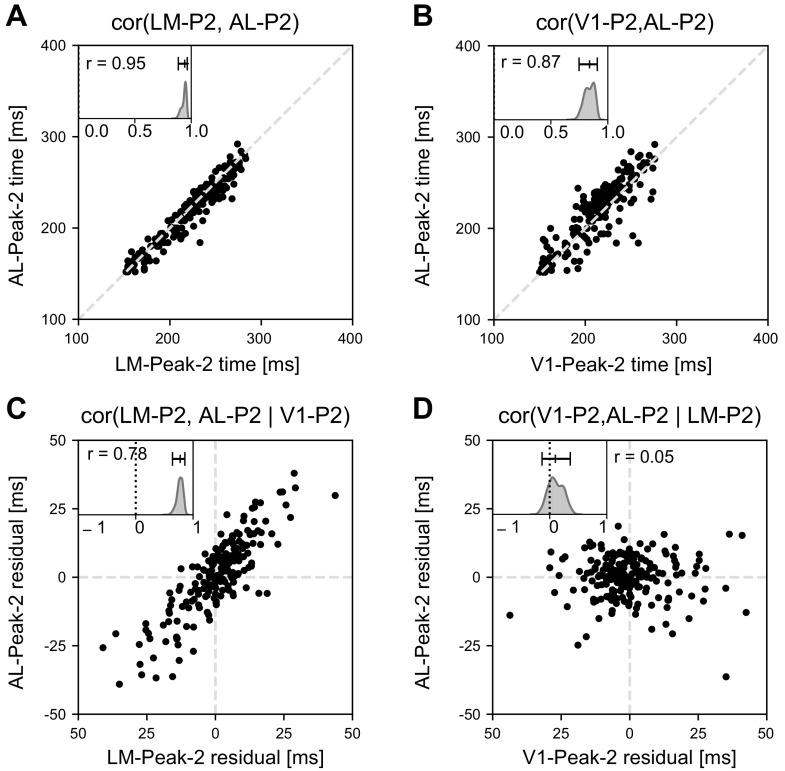
Peak-2 timing: correlation and partial correlation across regions. *A* and *B*: correlations, with each dot representing the estimated time of peak-2 on a given trial (using the posterior medians). *C* and *D*: partial correlation results, with the areas in *C* corresponding to those in *A* and the areas in *D* corresponding to those in *B*. In *C* and *D* each dot represents the residual from a regression on the conditioning variable (the correlation of these residuals being the partial correlation given the conditioning variable). All panels use posterior medians as estimates. *A*: despite large variability in peak-2 timing the correlation of lateral medial visual area (LM) peak-2 time and anterolateral visual area (AL) peak-2 time is close to 1. The plot embedded in the upper left corner displays the posterior distribution of the correlation [95% CI from 0.025 and 0.975 posterior quantiles (0.88, 0.96) with mode at 0.95]. *B*: this plot is for areas V1 and AL, analogous to that in *A* (95% CI from posterior quantiles (0.75, 0.91) with mode at 0.87). *C*: the residual Peak-2 times for LM and AL are plotted, after regressing on the Peak-2 time of V1 timing (95% CI from posterior quantiles (0.64,0.86) with mode at 0.78). *D*: the residual Peak-2 times for primary visual cortex (V1) and AL are plotted, after regressing on the Peak-2 time of LM. The partial correlation in *C* is somewhat smaller, but not dramatically smaller, than the correlation in *A*. In contrast, the partial correlation in *D* is close to zero, in sharp contrast to the large correlation in *B* [95% CI from posterior quantiles (−0.13, 0.36) with mode at 0.05].

We begin by discussing partial correlation results that might have been expected. In the feedforward case of Peak-1 times, one might expect that the time of Peak-1 in AL and LM was nearly wholly determined by the time of Peak-1 in V1. In that case, we would expect to find that the Peak-1 times in AL and LM are highly correlated, but the correlation largely disappears after conditioning on (statistically, as if holding fixed) the Peak-1 time in V1, leaving the partial correlation of Peak-1 times in AL and LM given the Peak-1 time in V1 statistically indistinguishable from zero. Because this would indicate the feedforward signal from V1 could account for the Peak-1 timing in the other two areas, it would constrain conceptions of circuit operation. However, this strict feedforward determination of timing is not what we found in our data, for either of the two peaks. In Fig. 6, we show results for Peak-2 times (see Supplemental Fig. S5 for additional results). In Fig. 6, *A* and *C* we see that, while the correlation between Peak-2 times in AL and LM were indeed highly correlated, after conditioning on the Peak-2 times in V1 the partial correlation decreased only a relatively small amount (the same was true for Peak-1 times). On the other hand, we found that conditioning on LM Peak-2 times greatly reduced the correlation of V1 and AL Peak-2 times, making it statistically indistinguishable from zero (Fig. 6, *B* and *D*).

Figure 6 demonstrates the potential power of examining multivariate coupling relationships among features of population firing rate functions. The results in Fig. 6*D* also indicate some circuit mechanism subtlety, especially in conjunction with Fig. 5*D*, which indicated that, for Peak-2, not only does V1 lead LM but AL tends to lead LM for the large majority of trials. That is, it is rare for the selected population of LM neurons to reach its Peak-2 maximal firing rate before that of the AL population. Furthermore, for Peak-2, as shown in Supplemental Fig. S5, we do not see such a dramatic reduction of the correlation of V1 and LM after conditioning on AL, nor do we see a dramatic reduction of the correlation of V1 and AL after conditioning on LM for Peak-1. We provide some further thoughts on interpretation of this result in the discussion. The important conclusion about the IPRF model is that it is capable of finding interesting three-way (or, more generally, multiway) relationships.

#### Population templates.

**Figure 7.**
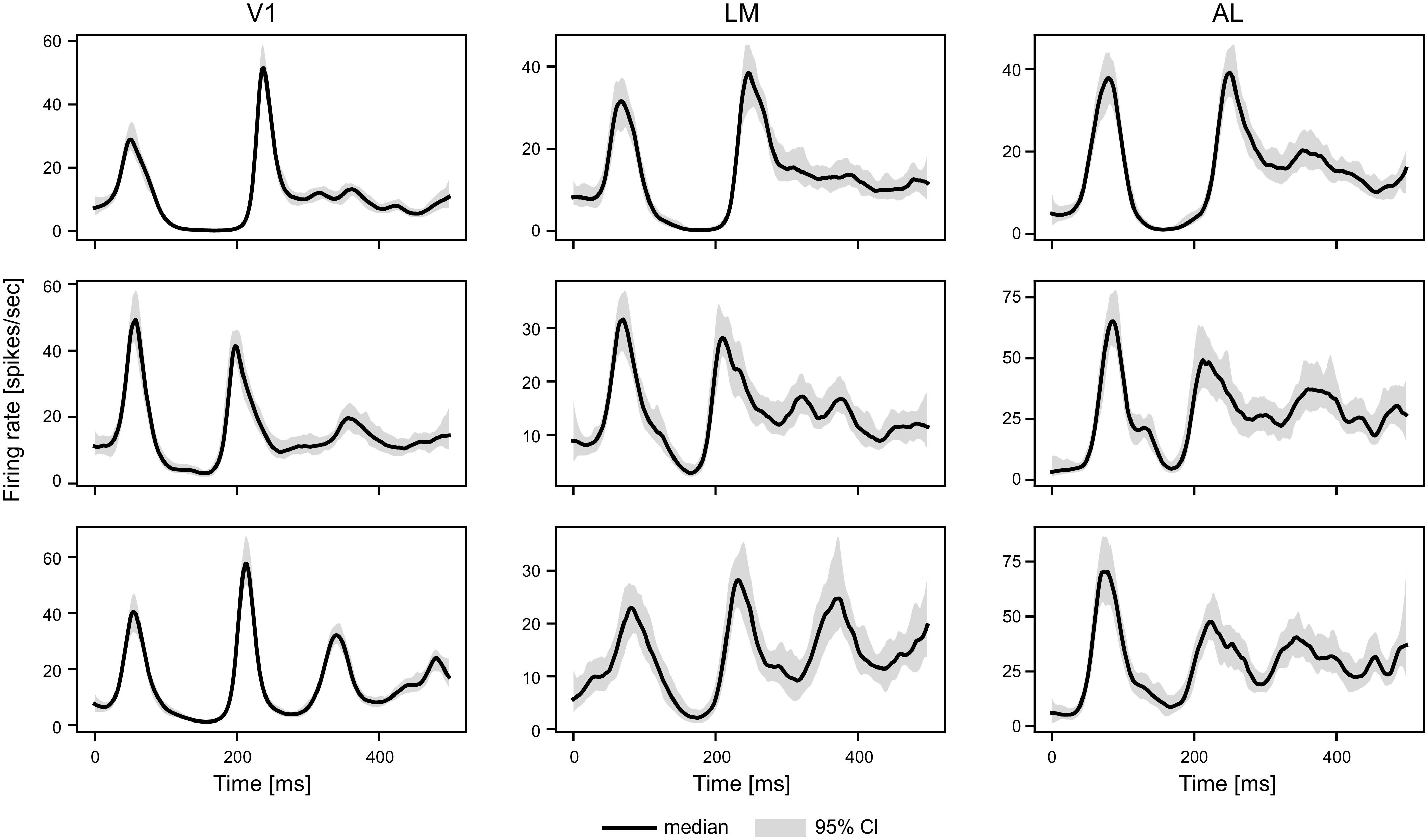
Fitted population firing rate templates. The figure shows population firing templates in three different conditions after exponentiating to get units of firing rate, i.e., for area *a* and condition *c* we plot $\exp\;\{f_{a,c}^{\text{pop}}\}$. In condition 281, the gratings drifted at 15 Hz with orientation 315°, in condition 257 at 8 Hz and 315°, and in condition 281 at 8 Hz and 270°. Note that the population firing rate function is $\exp\;\{N_{g=\text{pop},a,c}\cdot f_{a,c}^{\text{pop}}\}$ where *N_g_*_=pop,_*_a_*_,_*_c_* is the number of neurons for which *z_a,c_*(*n*) = pop. Each row represents one condition. The solid curves and gray bands are the medians and 95% CIs from the posteriors. There are striking distinctions between these firing rate curves and those in Fig. 1*A*. The complete set of curves is in Supplemental Fig. S8.

Figure 7 shows fitted population firing rate templates in three conditions, and Supplemental Fig. S8 shows the complete set. The V1 templates are very precisely determined (they have narrow posterior bands) and LM and AL templates are well determined. Our use of distinct templates for different conditions reveals both strong similarities and noticeable distinctions in the population responses across conditions. An important deviation in the fitted templates from the PSTH curves shown in Fig. 1*A* is that Peak-2 is taller and narrower in Fig. 7 than it appears in Fig. 1*A*. This is largely due to the trial-to-trial variation in peak time, which dampens Peak-2 in the PSTH. In addition, the much higher firing rates are also affected by the more refined selection of neurons in our model than in Fig. 1*A*. More details can be found in Supplemental Fig. S10.

#### Diversity of neurons.

**Figure 8.**
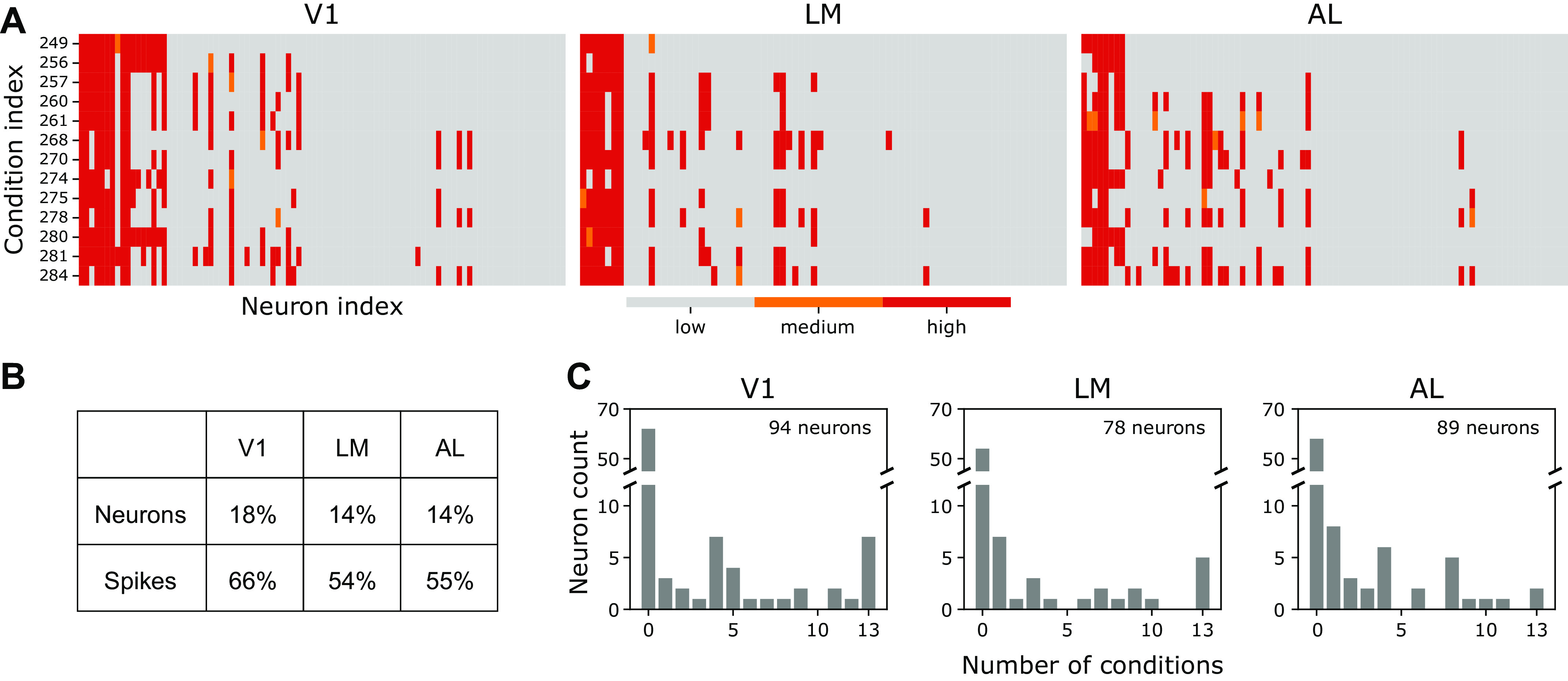
Neuron subpopulations. *A*: probability that a neuron is a member of the population having firing rate template $f_{a,c}^{\text{pop}}$, for all areas and conditions. Columns are distinct neurons, rows are different conditions: red indicates the probability is high (posterior median greater than 0.9); gray indicates the probability is low (posterior median less than 0.1); orange indicates an intermediate probability. *B*: proportion of activity contributing to the population represented by $f_{a,c}^{\text{pop}}$, averaged across conditions (based on the posterior medians in *A*). For example, on average, 18% of primary visual cortex (V1) neurons were part of the interacting population (with template $f_{a,c}^{\text{pop}}$), but they generated 66% of the spikes. *C*: histograms that summarize the plots in *A*. For each neuron, in each area *a*, we count the number of conditions *c* having posterior median of *p_a_*_,_*_c_*_;0_ greater than 0.9 (i.e., the number of red bars in the column corresponding to that neuron in *A*). The histograms display the number of neurons with count *x*, for *x* = 0,1,2,…,13. In each area, somewhat over half of the neurons never participated (*x* = 0) and those that did participate often participated in only a few conditions; in V1, 7 of the 94 neurons participated in all conditions. AL, anterolateral visual area; LM, lateral medial visual area.

Figure 8 summarizes the classification of neurons to interacting populations (“pop” neurons) that have templates *f*^pop^. As seen in Fig. 8*A*, for nearly all neurons it is clear whether they are fit better using the *f*^pop^ template or using one of *f*^local-1^ or *f*^local-2^. Only 22 out of 3,393 neuron-condition combinations fail to have at least 90% probability (from the posterior median) of one or the other. Figure 8*B* elaborates by showing how many neurons contribute, and what proportion of spikes they generate. Figure 8*C* indicates the diversity of condition-dependent neural responses. The large variation in individual-neuron responses is perhaps unsurprising, but it underscores the importance of allowing for such diversity in statistical modeling efforts. The portion of neurons in each subgroup (*p_a_*_,_*_c_* in *Eq. 9*) is shown in Supplemental Fig. S7. Supplemental Fig. S11 shows that there is no strong spatial pattern to neuron membership in the communicating population.

#### Goodness of fit.

**Figure 9.**
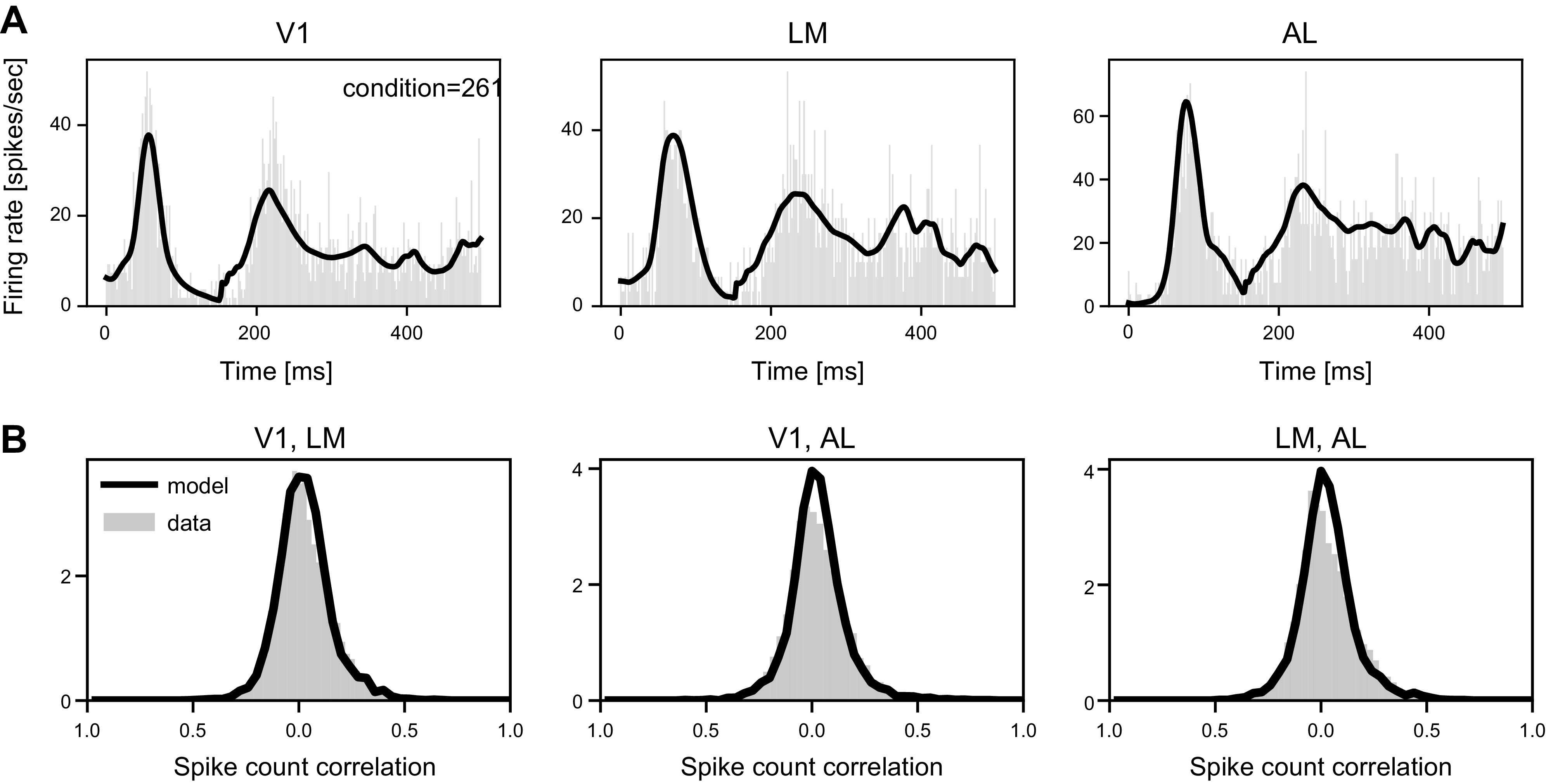
Model fits to peristimulus time histograms (PSTHs) and spike count correlation histograms. *A*: PSTHs for condition 261 (complete set is in Supplemental Fig. S14). The PSTHs are shown as gray histograms, and are constructed from the 15 trials for the condition, using the selected “pop” neurons. The dark curves are the fitted population intensity functions (λ*_g_*_,_*_a_*_,_*_r_*_,_*_c_*, as in *Eq. 1*, with *g* = pop) averaged across the 15 trials. *B*: histograms of spike count correlations, based on all pairs of “pop” neurons from the pair of areas, one neuron in the first area and one neuron in the second area, for the pairs in the labels at the top of each plot. Model fits, found from simulated spike trains, shown as dark curves. AL, anterolateral visual area; LM, lateral medial visual area.

The fitted IPRF model does a good job of capturing major spiking patterns in the data. Figure 9*A* shows examples of fits of the template functions to condition-specific PSTHs. The complete set of curves and more details can be found in Supplemental Fig. S14. Figure 9*B* shows fits to spike count correlations, and other cases are shown in Supplemental Fig. S15. Fits to cross-correlograms are shown in Supplemental Fig. S16. Population probability plots (P-P plots), also known as Kolmogorov–Smirnov or KS plots, appear in Supplemental Fig. S18.

### Simulations

The data we analyzed consisted of 15 trials per condition and, as shown in Fig. 8, the IPRF model frequently assigned as few as ∼10 neurons to the interacting population within a condition and area. Using similar sample sizes, we performed three types of simulations to assess performance. Simulation details are in Supplemental Simulation Study. In the first two types of simulations, we report results for correlations between features, which are the main interest (e.g., in Fig. 6).

First, we assumed the model structure is correct as described in *Eqs. 1–7* to check accuracy of coverage probability for putative 95% confidence intervals, and also to check the size of bias; in the small-bias case of classical asymptotic statistical theory, bias (of maximum likelihood estimators) is small compared to variance and is often ignored. Bias and variance are combined in mean squared error (which is the sum of variance and squared bias, and so its square root is on the scale of the estimate itself). We found small bias and small root mean squared error (RMSE). The bias values of the estimated correlations are in the range [–0.041, 0.040] (the mean of all pairs is −0.001). The posterior CIs are mostly close to their putative 95% coverage probability, though a few entries in the table of results are somewhat smaller (see Supplemental Table S3). The RMSE values are in range [0.022, 0.090] (the mean of all pairs is 0.072). The range of error is slightly larger than the lower bound of the estimation. See Supplemental Simulation Study for details. We also verified that simulation standard errors of CI end points were small relative to the RMSE values. The range of the values is [0.0016, 0.0091] and the mean standard error across all end points is 0.0056.

Our implementation of the IPRF model assumes that, for the purpose of peak timing identification, trial-to-trial variability in the population firing rate intensity function can be summarized as involving only shifts in peak timing. If time warping compensates for trial-varying changes in intensity shape, however, peak timing identification could be affected. Although one might expect such effects to be small, we did run a simple simulation study to check. To distort each trial’s population intensity, we injected noise into each neuron, in the form of intensity shapes that varied across neurons, which changes the population firing rate intensity function (see Supplemental Simulation Study). We chose the amount of injected neuron gain to roughly correspond to the real data. See methods. (As a check, we also tried a large noise setting with 20 times the variance.) We found that the covariation estimates are not very sensitive to this type of deviation from assumptions (see details in Supplemental Simulation Study and Tables S4 S5).

The final type of simulation involved different template shapes, shown in Supplemental Fig. S19. We examined not only a relatively sharp peak (panel *A*) similar to our data, but also a much broader peak (panel *B*), an inverted peak (panel *C*), and a firing rate increase to an asymptote (panel *D*). For the latter, the center of the template was taken to be half way up the incline to the maximum. In each case, our template matching scheme succeeded in locating the true position of the template, based on simulated data. These results suggest the IPRF approach should be applicable to many situations involving multiple population spike trains.

## DISCUSSION

The motivating goal of the IPRF model is to identify timing, variation, and covariation of transient, sharp increases in population firing rates. This is reminiscent of evoked response potential (ERP) analysis. Although evoked potential interpretations are based on trial averaging, being too noisy to be useful in single trials, the IPRF model is able to estimate denoised evoked population burst timing on a trial-by-trial basis. Accurate trial-by-trial estimation of population burst timing helps identify the putative propagation of information, supported by assessment of trial-by-trial covariation across areas, where peak coupling of pulsatile responses is similar in spirit to phase coupling of oscillations. The methods are not limited to analysis of evoked sensory responses. As we showed in *Simulations* and Supplemental Fig. S19, the methodology could be applied to population activity having a variety of temporal profiles as long as they have well-defined times at which a maximal firing rate (or minimal firing rate) can be said to occur. In fact, some version of the same idea should also be applicable to deconvolved spike trains obtained from calcium imaging.

For our data analytic context, we applied the powerful IPRF machinery to analysis of the two peaks in the visual responses in mouse visual areas V1, AL, and LM, seen in Fig. 1. We expected to see some distinctions in our analyses of the two peaks because Peak-1 timing, early in the response, should be driven by feed forward activity, whereas Peak-2 timing would involve both feed forward and feedback signals. The variation across trials in Peak-1 timing may conform roughly to intuition: for estimated mean response delays ranging from 57 to 70 ms after stimulus onset, we found the standard deviation to be around 5 ms for V1 and AL and 7.5 ms for LM (see Fig. 5*A*). On the other hand, the variation in Peak-2 timing is large. According to our results (Fig. 5*C*), for estimated mean response delays ranging from 216 to 233 ms, the standard deviations are roughly 30 ms. In this context, it was remarkable to find the very large correlations in Peak-2 timing across areas (lower end of confidence intervals greater than 0.75; results were confirmed in a second mouse, with somewhat smaller correlations; and correlations were higher for Peak-2 than for Peak-1, see Supplemental Fig. S5). The results show there are populations of cells in V1, AL, and LM whose delayed responses to stimuli are all modulated by trial-to-trial variation in very much the same way. Although it is not possible to draw causal conclusions from correlations, it is reasonable to expect that trials on which a peak population firing rate occurs relatively early would also be trials on which population transmission of information occurs relatively early. The high correlation in Peak-2 timing, together with low-variation lag times (standard deviations of roughly 2.5 ms; Fig. 5*D*), suggests the visual processing network that includes V1, AL, and LM is tightly coordinated on a trial-by-trial basis, even in the presence of large trial-by-trial (“noise”) variation.

Several scientific caveats should be emphasized. Population peak coupling identifies a form of what is usually called functional connectivity. When we say that population peak coupling reveals coordinated activity, we do not mean that it must be purposeful in a mechanistic sense. For one thing, only a few brain areas are being analyzed, and in any setting there are bound to be complicated anatomical connectivity patterns, as there are for the mouse visual system (2, 19). But even if we were able to observe all areas that are relevant to a given task, aside from special circumstances causal relationships could only be established from experiments using causal interventions (43–45). Moreover, while features such as peak timing are useful, they offer a limited description of interaction dynamics. In addition, despite the new capabilities provided by recording arrays such as Neuropixels, the neurons recorded from any area do not constitute a random sample of all relevant neurons, nor are the neuron types identified. With current experimental data, the extent to which recording creates important biases remains unknown, and conclusions must remain cautious. For example, the dramatic reduction in correlation of V1 and AL Peak-2 timing after conditioning on LM Peak-2 timing (also replicated in the second mouse, Supplemental Fig. S12) may occur because LM provides important feedback input to AL that enhances its Peak-2 rise time, and this might also help explain the much shorter lags from the maximal firing rate in V1 to those in AL and LM for Peak-2 than for Peak-1. However, other areas might provide such input to both AL and LM. Furthermore, if we were to ignore feedback and take the relative timing of the template peaks as conclusive, in conjunction with our partial correlation results, we might infer directional relations V1 → AL → LM combined with V1 → LM. All of these possible transmission pathways may well be operative, together, and we are not offering evidence for specific scientific hypotheses. Our purpose here is to show that investigations of population burst timing across brain areas can generate potentially interesting findings, which would have to be explainable by future conceptions of circuit operation. Other intriguing patterns may be found in Supplemental Figs. S3, S4, and S5 and Supplemental Table S2.

Statistically, our initialization procedure, while straightforward, was crucial. In some other situations it might be more challenging. Also, we assumed that a single population of neurons was most relevant for both Peak-1 and Peak-2. It is possible that treating each peak separately would be advantageous. More fundamentally, we have assumed that trial-to-trial variation in population burst timing can be captured accurately with a model in which timing of maximal firing rate, and gain (time-averaged firing rate), are the only sources of trial-to-trial variation. Surely there are other ways that firing rate intensities vary across trials, and our simulation study checked consequences for one type of noncompliant variation. For the accuracy of our conclusions, what matters is whether noncompliant effects produce substantially different times at which the peaks occur and substantially different correlations of peak timing. The flexibility of the IPRF model allows the time of maximal firing to be relatively well determined, statistically, from the large number of spikes surrounding that time, and we would not expect it to be sensitive to minor model misspecifications.

We are not aware of any existing data analytic methods that could produce the conclusions reached from the IPRF model. Figure 1*A* shows that naive curve fitting is inadequate, and dimensionality reduction methods are unlikely to solve this problem: we tried applying Gaussian Process Factor Analysis (GPFA; 46, 13) but that, again, produced a useless plot, much like Fig. 1*A* (see Supplemental Fig. S6). Nor were we able to obtain from GPFA the kind of detailed assessment of timing relationships across areas we obtained with the IPRF model.

The implementation we reported here involves a comprehensive Bayesian hierarchical model. It would be possible to decompose some of the components in our IPRF model while also accounting for important sources of variation in different steps (47). As we described, however, it was straightforward to implement EM and Gibbs sampling for the comprehensive model, which has the advantage of making it easy to get an assessment of uncertainty for any model-based quantity we wish to estimate. We hope our work will stimulate further efforts to harness the power of point process modeling for investigating timing relationships among neural populations and their coordination across brain areas.

## SUPPLEMENTAL DATA

10.5281/zenodo.7024023 Supplemental Material: https://www.doi.org/10.5281/zenodo.7024023.

## GRANTS

B.J.M. and H.D. were supported as undergraduates on National Institute of Drug Abuse training Grant T90 DA022762. Y.C. was supported in part by T90 DA022762 and in part by National Institute of Mental Health Grant R01 MH064537. R.E.K. and M.O. were supported on R01 MH064537.

## DISCLOSURES

No conflicts of interest, financial or otherwise, are declared by the authors.

## AUTHOR CONTRIBUTIONS

Y.C., J.H.S., and R.E.K. conceived and designed research; J.H.S. performed experiments; Y.C., H.D., B.J.M., and M.O. analyzed data; Y.C., J.H.S., and R.E.K. interpreted results of experiments; Y.C. prepared figures; Y.C. and R.E.K. drafted manuscript; Y.C., H.D., B.J.M., M.O., J.H.S., and R.E.K. edited and revised manuscript; Y.C., H.D., B.J.M., M.O., J.H.S., and R.E.K. approved final version of manuscript.

## ENDNOTE

At the request of the authors, readers are herein alerted to the fact that additional materials related to this manuscript may be found at www.github.com/AlbertYuChen/IPRF. These materials are not a part of this manuscript and have not undergone peer review by the American Physiological Society (APS). APS and the journal editors take no responsibility for these materials, for the website address, or for any links to or from it.

## References

[1] Jun JJ, Steinmetz NA, Siegle JH, Denman DJ, Bauza M, Barbarits B, Lee AK, Anastassiou CA, Andrei A, Aydın Ç, Barbic M, Blanche TJ, Bonin V, Couto J, Dutta B, Gratiy SL, Gutnisky DA, Häusser M, Karsh B, Ledochowitsch P, Lopez CM, Mitelut C, Musa S, Okun M, Pachitariu M, Putzeys J, Rich PD, Rossant C, Sun W-L, Svoboda K, Carandini M, Harris KD, Koch C, O'Keefe J, Harris TD. Fully integrated silicon probes for high-density recording of neural activity. Nature 551: 232–236, 2017. doi:10.1038/nature24636. 29120427 PMC5955206

[2] Siegle JH, Jia X, Durand S, Gale S, Bennett C, Graddis N, Heller G, Ramirez TK, Choi H, Luviano JA, Groblewski PA, Ahmed R, Arkhipov A, Bernard A, Billeh YN, Brown D, Buice MA, Cain N, Caldejon S, Casal L, Cho A, Chvilicek M, Cox TC, Dai K, Denman DJ, de Vries SEJ, Dietzman R, Esposito L, Farrell C, Feng D. Survey of spiking in the mouse visual system reveals functional hierarchy. Nature 592: 86–92, 2021. doi:10.1038/s41586-020-03171-x. 33473216 PMC10399640

[3] Steinmetz NA, Aydin C, Lebedeva A, Okun M, Pachitariu M, Bauza M, Beau M, Bhagat J, Böhm C, Broux M, Chen S, Colonell J, Gardner RJ, Karsh B, Kloosterman F, Kostadinov D, Mora-Lopez C, O’Callaghan J, Park J, Putzeys J, Sauerbrei B, van Daal RJJ, Vollan AZ, Wang S, Welkenhuysen M, Ye Z, Dudman JT, Dutta B, Hantman AW, Harris KD. Neuropixels 2.0: a miniaturized high-density probe for stable, long-term brain recordings. Science 372: eabf4588, 2021. doi:10.1126/science.abf4588. 33859006 PMC8244810

[4] Ben-Shaul Y, Bergman H, Ritov Y, Abeles M. Trial to trial variability in either stimulus or action causes apparent correlation and synchrony in neuronal activity. J Neurosci Methods 111: 99–110, 2001. doi:10.1016/s0165-0270(01)00389-2. 11595277

[5] Brody CD. Disambiguating different covariation types. Neural Comput 11: 1527–1535, 1999. doi:10.1162/089976699300016124. 10490936

[6] Ventura V, Cai C, Kass RE. Trial-to-trial variability and its effect on time-varying dependency between two neurons. J Neurophysiol 94: 2928–2939, 2005. doi:10.1152/jn.00644.2004. 16160096

[7] Averbeck BB, Latham PE, Pouget A. Neural correlations, population coding and computation. Nat Rev Neurosci 7: 358–366, 2006. doi:10.1038/nrn1888. 16760916

[8] Cohen MR, Kohn A. Measuring and interpreting neuronal correlations. Nat Neurosci 14: 811–819, 2011. doi:10.1038/nn.2842. 21709677 PMC3586814

[9] Glaser J, Whiteway M, Cunningham JP, Paninski L, Linderman S. Recurrent switching dynamical systems models for multiple interacting neural populations. Advances in Neural Information Processing Systems 33, edited by Larochelle H, Ranzato M, Hadsell R, Balcan MF, Lin H. NeurIPS 2020, 2020, p. 14867–14878.

[10] Gokcen E, Jasper AI, Semedo JD, Zandvakili A, Kohn A, Machens CK, Yu BM. Disentangling the flow of signals between populations of neurons (Preprint). bioRxiv, 2021. doi:10.1101/2021.08.30.458230. PMC11442031 38177794

[11] Semedo JD, Gokcen E, Machens CK, Kohn A, Yu BM. Statistical methods for dissecting interactions between brain areas. Curr Opin Neurobiol 65: 59–69, 2020. doi:10.1016/j.conb.2020.09.009. 33142111 PMC7935404

[12] Semedo JD, Jasper AI, Zandvakili A, Krishna A, Aschner A, Machens CK, Kohn A, Yu BM. Feedforward and feedback interactions between visual cortical areas use different population activity patterns. Nat Commun 13: 1099, 2022. doi:10.1038/s41467-022-28552-w. 35232956 PMC8888615

[13] Yu BM, Cunningham JP, Santhanam G, Ryu SI, Shenoy KV, Sahani M. Gaussian-process factor analysis for low-dimensional single-trial analysis of neural population activity. J Neurophysiol 102: 614–635, 2009 [Erratum in *J Neurophysiol* 102: 2008, 2009]. doi:10.1152/jn.90941.2008. 19357332 PMC2712272

[14] Keeley S, Aoi M, Yu Y, Smith S, Pillow JW. Identifying signal and noise structure in neural population activity with gaussian process factor models. Advances in Neural Information Processing Systems 33, edited by Larochelle H, Ranzato M, Hadsell R, Balcan MF, Lin H. NeurIPS 2020, 2020, p. 13795–13805.

[15] Duncker L, Sahani M. Temporal alignment and latent Gaussian process factor inference in population spike trains. Advances in Neural Information Processing Systems 31, edited by Bengio S, Wallach H, Larochelle H, Grauman K, Cesa-Bianchi N, Garnett R. NeurIPS 2018, 2018.

[16] Eberstein A, Sandow A. Fatigue in phasic and tonic fibers of frog muscle. Science 134: 383–384, 1961. doi:10.1126/science.134.3476.383. 13725552

[17] Grace AA. Phasic versus tonic dopamine release and the modulation of dopamine system responsivity: a hypothesis for the etiology of schizophrenia. Neuroscience 41: 1–24, 1991. doi:10.1016/0306-4522(91)90196-u. 1676137

[18] Allen Institute for Brain Science. Visual Coding—Neuropixels. https://portal.brain-map.org/explore/circuits/visual-coding-neuropixels [2022 Nov 23].

[19] Harris JA, Mihalas S, Hirokawa KE, Whitesell JD, Choi H, Bernard A, Bohn P, Caldejon S, Casal L, Cho A, Feiner A, Feng D, Gaudreault N, Gerfen CR, Graddis N, Groblewski PA, Henry AM, Ho A, Howard R, Knox JE, Kuan L, Kuang X, Lecoq J, Lesnar P, Li Y, Luviano J, McConoughey S, Mortrud MT, Naeemi M, Ng L. Hierarchical organization of cortical and thalamic connectivity. Nature 575: 195–202, 2019. doi:10.1038/s41586-019-1716-z. 31666704 PMC8433044

[20] Del Cul A, Baillet S, Dehaene S. Brain dynamics underlying the nonlinear threshold for access to consciousness. PLoS Biol 5: e260, 2007. doi:10.1371/journal.pbio.0050260. 17896866 PMC1988856

[21] Yang Y, Tarr MJ, Kass RE, Aminoff EM. Exploring spatiotemporal neural dynamics of the human visual cortex. Hum Brain Mapp 40: 4213–4238, 2019. doi:10.1002/hbm.24697. 31231899 PMC6865718

[22] Supèr H, Spekreijse H, Lamme VA. Two distinct modes of sensory processing observed in monkey primary visual cortex (v1). Nat Neurosci 4: 304–310, 2001. doi:10.1038/85170. 11224548

[23] Manita S, Suzuki T, Homma C, Matsumoto T, Odagawa M, Yamada K, Ota K, Matsubara C, Inutsuka A, Sato M, Ohkura M, Yamanaka A, Yanagawa Y, Nakai J, Hayashi Y, Larkum ME, Murayama M. A top-down cortical circuit for accurate sensory perception. Neuron 86: 1304–1316, 2015. doi:10.1016/j.neuron.2015.05.006. 26004915

[24] Sachidhanandam S, Sreenivasan V, Kyriakatos A, Kremer Y, Petersen CC. Membrane potential correlates of sensory perception in mouse barrel cortex. Nat Neurosci 16: 1671–1677, 2013. doi:10.1038/nn.3532. 24097038

[25] Behseta S, Berdyyeva T, Olson CR, Kass RE. Bayesian correction for attenuation of correlation in multi-trial spike count data. J Neurophysiol 101: 2186–2193, 2009. doi:10.1152/jn.90727.2008. 19129297 PMC2695642

[26] Vinci G, Ventura V, Smith MA, Kass RE. Separating spike count correlation from firing rate correlation. Neural Comput 28: 849–881, 2016. doi:10.1162/NECO_a_00831. 26942746 PMC4842091

[27] Keeley S, Zoltowski DM, Aoi MC, Pillow JW. Modeling statistical dependencies in multi-region spike train data. Curr Opin Neurobiol 65: 194–202, 2020. doi:10.1016/j.conb.2020.11.005. 33334641 PMC7769979

[28] Kramer MA, Eden UT. Case Studies in Neural Data Analysis: A Guide for the Practicing Neuroscientist. Cambridge, MA: MIT Press, 2016.

[29] Pillow JW, Shlens J, Paninski L, Sher A, Litke AM, Chichilnisky EJ, Simoncelli EP. Spatio-temporal correlations and visual signalling in a complete neuronal population. Nature 454: 995–999, 2008. doi:10.1038/nature07140. 18650810 PMC2684455

[30] Truccolo W, Eden UT, Fellows MR, Donoghue JP, Brown EN. A point process framework for relating neural spiking activity to spiking history, neural ensemble, and extrinsic covariate effects. J Neurophysiol 93: 1074–1089, 2005. doi:10.1152/jn.00697.2004. 15356183

[31] Kass RE, Eden UT, Brown EN. Analysis of Neural Data. New York: Springer, 2014.

[32] Glickfeld LL, Olsen SR. Higher-order areas of the mouse visual cortex. Annu Rev Vis Sci 3: 251–273, 2017. doi:10.1146/annurev-vision-102016-061331. 28746815

[33] Marshel JH, Garrett ME, Nauhaus I, Callaway EM. Functional specialization of seven mouse visual cortical areas. Neuron 72: 1040–1054, 2011. doi:10.1016/j.neuron.2011.12.004. 22196338 PMC3248795

[34] Niell CM, Stryker MP. Highly selective receptive fields in mouse visual cortex. J Neurosci 28: 7520–7536, 2008. doi:10.1523/JNEUROSCI.0623-08.2008. 18650330 PMC3040721

[35] Murphy KP. Machine Learning: a Probabilistic Perspective. Cambridge, MA: MIT Press, 2012.

[36] Pouzat C, Chaffiol A. Automatic spike train analysis and report generation. an implementation with r, r2html and star. J Neurosci Methods 181: 119–144, 2009. doi:10.1016/j.jneumeth.2009.01.037. 19473708

[37] Silverman BW. Spline smoothing: the equivalent variable kernel method. Ann Stat 12: 898–916, 1984. doi:10.1214/aos/1176346710.

[38] Hastie T, Tibshirani R, Friedman J. The Elements of Statistical Learning: Data Mining, Inference, and Prediction. New York: Springer Science & Business Media, 2009.

[39] Gelman A, Carlin JB, Stern HS, Dunson DB, Vehtari A, Rubin DB. Bayesian Data Analysis. Boca Raton, FL: CRC Press, 2013.

[40] Boyd S, Boyd SP, Vandenberghe L. Convex Optimization. Cambridge, UK: Cambridge University Press, 2004.

[41] Brown EN, Barbieri R, Ventura V, Kass RE, Frank LM. The time-rescaling theorem and its application to neural spike train data analysis. Neural Comput 14: 325–346, 2002. doi:10.1162/08997660252741149. 11802915

[42] Haslinger R, Pipa G, Brown E. Discrete time rescaling theorem: determining goodness of fit for discrete time statistical models of neural spiking. Neural Comput 22: 2477–2506, 2010. doi:10.1162/NECO_a_00015. 20608868 PMC2932849

[43] Marinescu IE, Lawlor PN, Kording KP. Quasi-experimental causality in neuroscience and behavioural research. Nat Hum Behav 2: 891–898, 2018. doi:10.1038/s41562-018-0466-5. 30988445

[44] Richardson TS, Rotnitzky A. Causal etiology of the research of James M. Robins. Stat Sci 29: 459–484, 2014. doi:10.1214/14-STS505.

[45] Robins JM, Wasserman L. On the impossibility of inferring causation from association without background knowledge. In: Computation, Causation, and Discovery, edited by Glymour C, Cooper G. Cambridge, MA: MIT Press, 1999, p. 305–321.

[46] Lakshmanan KC, Sadtler PT, Tyler-Kabara EC, Batista AP, Yu BM. Extracting low-dimensional latent structure from time series in the presence of delays. Neural Comput 27: 1825–1856, 2015. doi:10.1162/NECO_a_00759. 26079746 PMC4545403

[47] Daniels MJ, Kass RE. A note on first-stage approximation in two-stage hierarchical models. Sankhyā Indian J Stat Ser B 60: 19–30, 1998.

